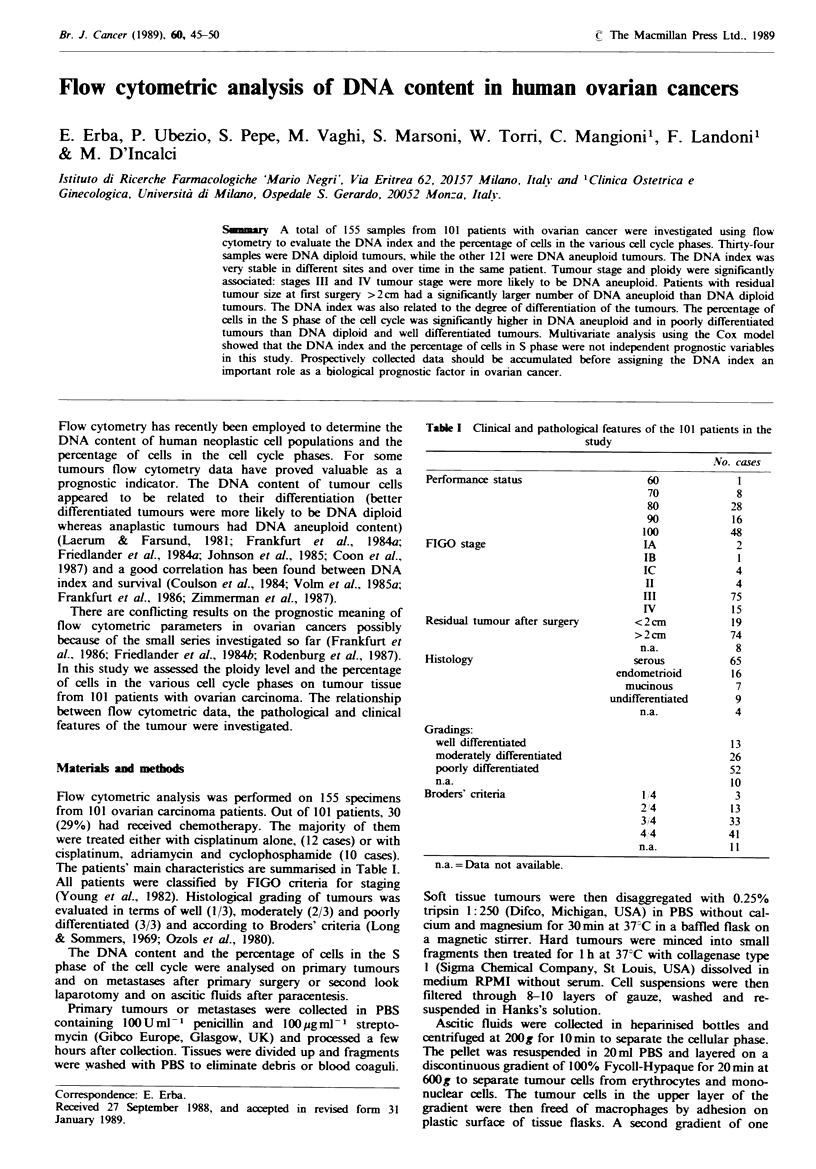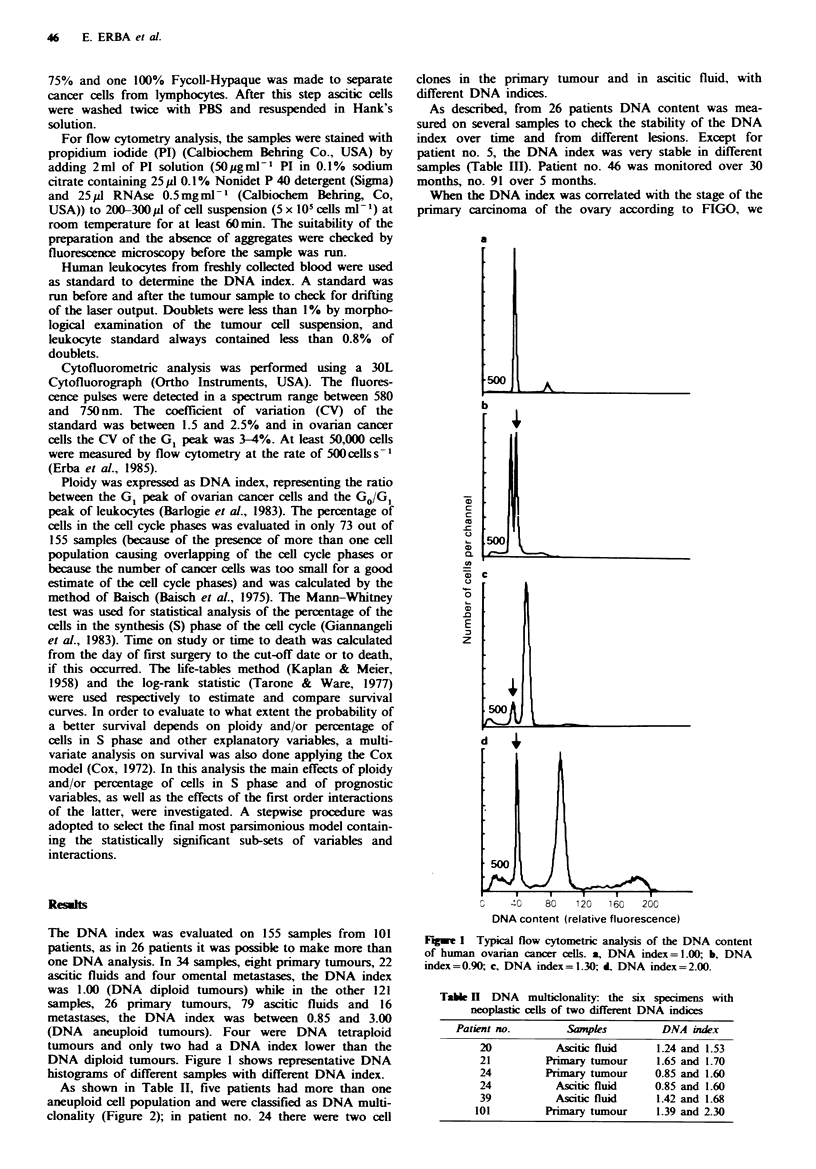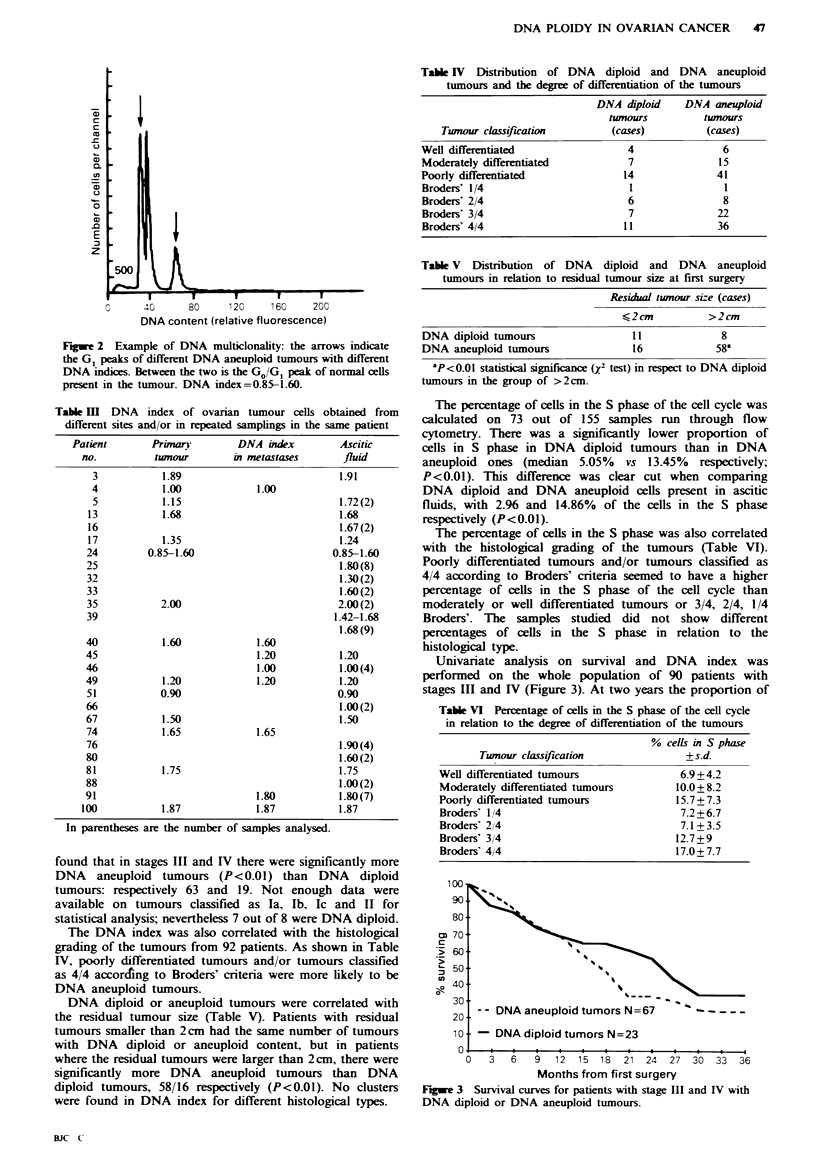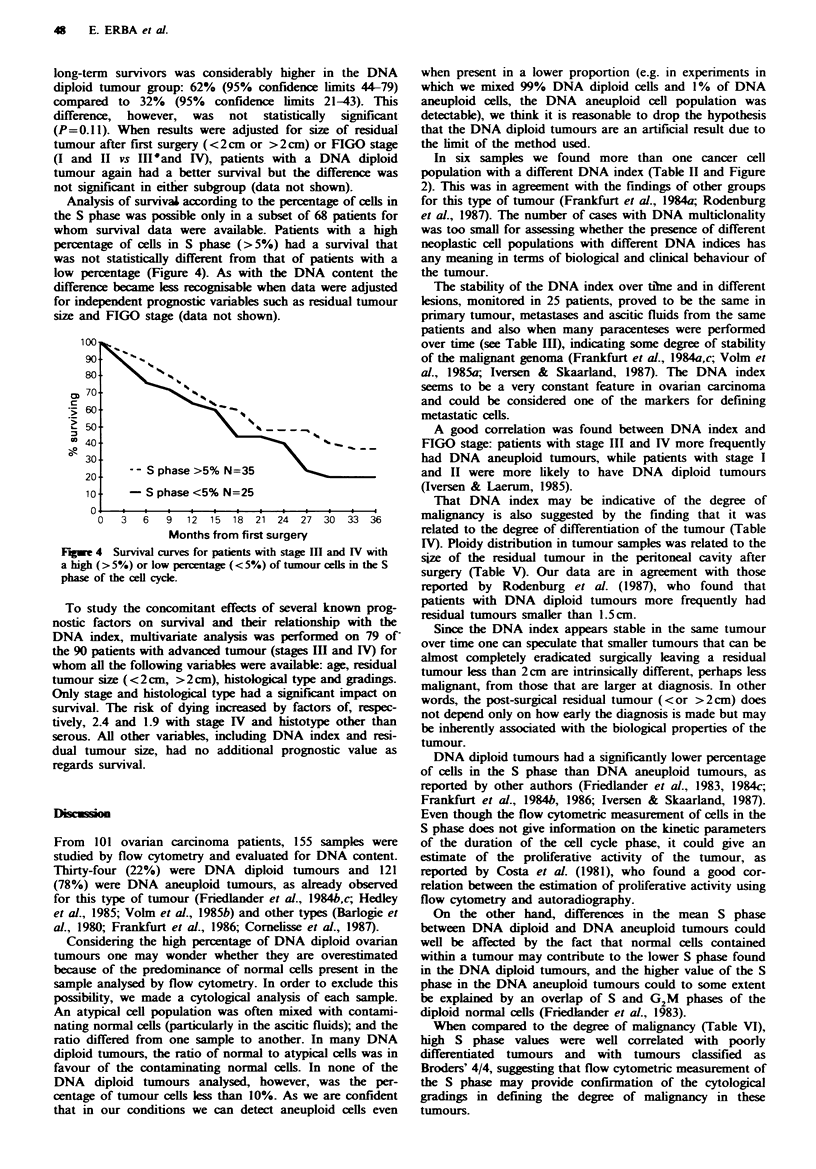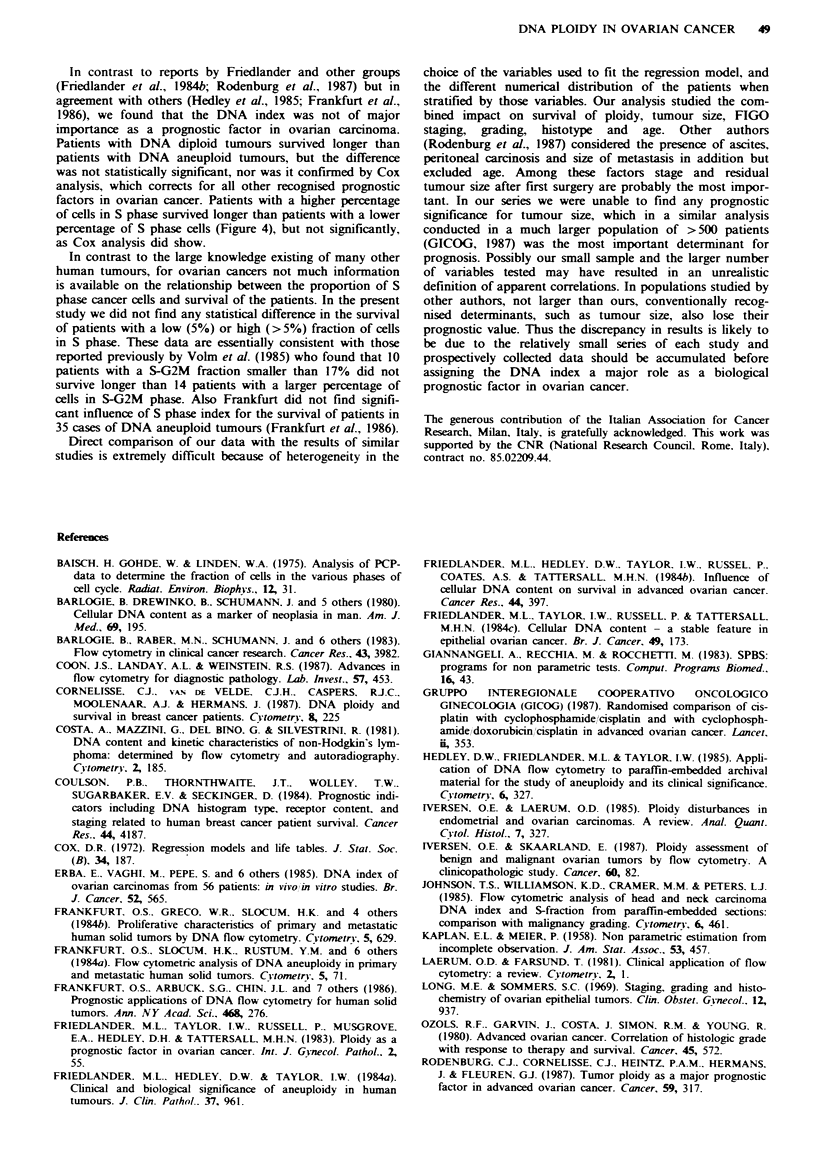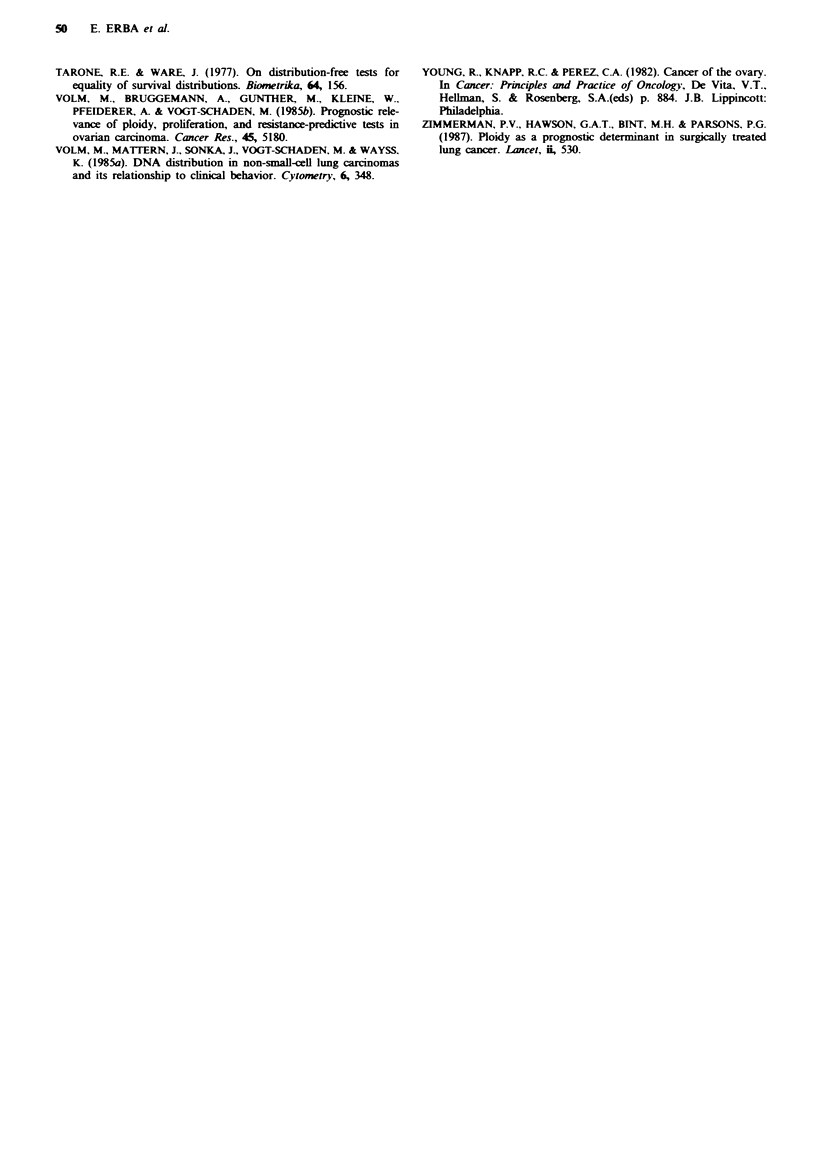# Flow cytometric analysis of DNA content in human ovarian cancers.

**DOI:** 10.1038/bjc.1989.217

**Published:** 1989-07

**Authors:** E. Erba, P. Ubezio, S. Pepe, M. Vaghi, S. Marsoni, W. Torri, C. Mangioni, F. Landoni, M. D'Incalci

**Affiliations:** Istituto di Ricerche Farmacologiche Mario Negri, Milano, Italy.

## Abstract

A total of 155 samples from 101 patients with ovarian cancer were investigated using flow cytometry to evaluate the DNA index and the percentage of cells in the various cell cycle phases. Thirty-four samples were DNA diploid tumours, while the other 121 were DNA aneuploid tumours. The DNA index was very stable in different sites and over time in the same patient. Tumour stage and ploidy were significantly associated: stages III and IV tumour stage were more likely to be DNA aneuploid. Patients with residual tumour size at first surgery greater than 2 cm had a significantly larger number of DNA aneuploid than DNA diploid tumours. The DNA index was also related to the degree of differentiation of the tumours. The percentage of cells in the S phase of the cell cycle was significantly higher in DNA aneuploid and in poorly differentiated tumours than DNA diploid and well differentiated tumours. Multivariate analysis using the Cox model showed that the DNA index and the percentage of cells in S phase were not independent prognostic variables in this study. Prospectively collected data should be accumulated before assigning the DNA index an important role as a biological prognostic factor in ovarian cancer.


Br. J. Cancer (1989), 60, 45-50 © The Macmillan Press Ltd., 1989

### Flow cytometric analysis of DNA content in human ovarian cancers

E. Erba, P. Ubezio, S. Pepe, M. Vaghi, S. Marsoni, W. Torri, C. Mangioni[1], F. Landoni[1] & M. D'Incalci

*Istituto di Ricerche Farmacologiche 'Mario Negri', Via Eritrea 62, 20157 Milano, Italy and [1]Clinica Ostetrica e Ginecologica, Università di Milano, Ospedale S. Gerardo, 20052 Monza, Italy.*

**Summary** A total of 155 samples from 101 patients with ovarian cancer were investigated using flow cytometry to evaluate the DNA index and the percentage of cells in the various cell cycle phases. Thirty-four samples were DNA diploid tumours, while the other 121 were DNA aneuploid tumours. The DNA index was very stable in different sites and over time in the same patient. Tumour stage and ploidy were significantly associated: stages III and IV tumour stage were more likely to be DNA aneuploid. Patients with residual tumour size at first surgery >2cm had a significantly larger number of DNA aneuploid than DNA diploid tumours. The DNA index was also related to the degree of differentiation of the tumours. The percentage of cells in the S phase of the cell cycle was significantly higher in DNA aneuploid and in poorly differentiated tumours than DNA diploid and well differentiated tumours. Multivariate analysis using the Cox model showed that the DNA index and the percentage of cells in S phase were not independent prognostic variables in this study. Prospectively collected data should be accumulated before assigning the DNA index an important role as a biological prognostic factor in ovarian cancer.

Flow cytometry has recently been employed to determine the DNA content of human neoplastic cell populations and the percentage of cells in the cell cycle phases. For some tumours flow cytometry data have proved valuable as a prognostic indicator. The DNA content of tumour cells appeared to be related to their differentiation (better differentiated tumours were more likely to be DNA diploid whereas anaplastic tumours had DNA aneuploid content) (Laerum & Farsund, 1981; Frankfurt *et al.*, 1984*a*; Friedlander *et al.*, 1984*a*; Johnson *et al.*, 1985; Coon *et al.*, 1987) and a good correlation has been found between DNA index and survival (Coulson *et al.*, 1984; Volm *et al.*, 1985*a*; Frankfurt *et al.*, 1986; Zimmerman *et al.*, 1987).

There are conflicting results on the prognostic meaning of flow cytometric parameters in ovarian cancers possibly because of the small series investigated so far (Frankfurt *et al.*, 1986; Friedlander *et al.*, 1984*b*; Rodenburg *et al.*, 1987). In this study we assessed the ploidy level and the percentage of cells in the various cell cycle phases on tumour tissue from 101 patients with ovarian carcinoma. The relationship between flow cytometric data, the pathological and clinical features of the tumour were investigated.

### Materials and methods

Flow cytometric analysis was performed on 155 specimens from 101 ovarian carcinoma patients. Out of 101 patients, 30 (29%) had received chemotherapy. The majority of them were treated either with cisplatinum alone, (12 cases) or with cisplatinum, adriamycin and cyclophosphamide (10 cases). The patients' main characteristics are summarised in Table I. All patients were classified by FIGO criteria for staging (Young *et al.*, 1982). Histological grading of tumours was evaluated in terms of well (1/3), moderately (2/3) and poorly differentiated (3/3) and according to Broders' criteria (Long & Sommers, 1969; Ozols *et al.*, 1980).

The DNA content and the percentage of cells in the S phase of the cell cycle were analysed on primary tumours and on metastases after primary surgery or second look laparotomy and on ascitic fluids after paracentesis.

Primary tumours or metastases were collected in PBS containing 100 U ml$^{-1}$ penicillin and 100 $\mu$g ml$^{-1}$ streptomycin (Gibco Europe, Glasgow, UK) and processed a few hours after collection. Tissues were divided up and fragments were washed with PBS to eliminate debris or blood coaguli.

Correspondence: E. Erba.
Received 27 September 1988, and accepted in revised form 31 January 1989.

**Table I** Clinical and pathological features of the 101 patients in the study

| | | *No. cases* |
|---|---|---|
| Performance status | 60 | 1 |
| | 70 | 8 |
| | 80 | 28 |
| | 90 | 16 |
| | 100 | 48 |
| FIGO stage | IA | 2 |
| | IB | 1 |
| | IC | 4 |
| | II | 4 |
| | III | 75 |
| | IV | 15 |
| Residual tumour after surgery | <2 cm | 19 |
| | >2 cm | 74 |
| | n.a. | 8 |
| Histology | serous | 65 |
| | endometrioid | 16 |
| | mucinous | 7 |
| | undifferentiated | 9 |
| | n.a. | 4 |
| Gradings: | | |
| well differentiated | | 13 |
| moderately differentiated | | 26 |
| poorly differentiated | | 52 |
| n.a. | | 10 |
| Broders' criteria | 1/4 | 3 |
| | 2/4 | 13 |
| | 3/4 | 33 |
| | 4/4 | 41 |
| | n.a. | 11 |

n.a. = Data not available.

Soft tissue tumours were then disaggregated with 0.25% tripsin 1:250 (Difco, Michigan, USA) in PBS without calcium and magnesium for 30 min at 37°C in a baffled flask on a magnetic stirrer. Hard tumours were minced into small fragments then treated for 1 h at 37°C with collagenase type 1 (Sigma Chemical Company, St Louis, USA) dissolved in medium RPMI without serum. Cell suspensions were then filtered through 8-10 layers of gauze, washed and resuspended in Hanks's solution.

Ascitic fluids were collected in heparinised bottles and centrifuged at 200 g for 10 min to separate the cellular phase. The pellet was resuspended in 20 ml PBS and layered on a discontinuous gradient of 100% Fycoll-Hypaque for 20 min at 600 g to separate tumour cells from erythrocytes and mononuclear cells. The tumour cells in the upper layer of the gradient were then freed of macrophages by adhesion on plastic surface of tissue flasks. A second gradient of one

75% and one 100% Fycoll-Hypaque was made to separate cancer cells from lymphocytes. After this step ascitic cells were washed twice with PBS and resuspended in Hank's solution.

For flow cytometry analysis, the samples were stained with propidium iodide (PI) (Calbiochem Behring Co., USA) by adding 2 ml of PI solution ($50\,\mu g\,ml^{-1}$ PI in 0.1% sodium citrate containing $25\,\mu l$ 0.1% Nonidet P 40 detergent (Sigma) and $25\,\mu l$ RNAse $0.5\,mg\,ml^{-1}$ (Calbiochem Behring, Co, USA)) to 200–300 $\mu l$ of cell suspension ($5\times10^5$ cells $ml^{-1}$) at room temperature for at least 60 min. The suitability of the preparation and the absence of aggregates were checked by fluorescence microscopy before the sample was run.

Human leukocytes from freshly collected blood were used as standard to determine the DNA index. A standard was run before and after the tumour sample to check for drifting of the laser output. Doublets were less than 1% by morphological examination of the tumour cell suspension, and leukocyte standard always contained less than 0.8% of doublets.

Cytofluorometric analysis was performed using a 30L Cytofluorograph (Ortho Instruments, USA). The fluorescence pulses were detected in a spectrum range between 580 and 750 nm. The coefficient of variation (CV) of the standard was between 1.5 and 2.5% and in ovarian cancer cells the CV of the $G_1$ peak was 3–4%. At least 50,000 cells were measured by flow cytometry at the rate of 500 cells $s^{-1}$ (Erba *et al.*, 1985).

Ploidy was expressed as DNA index, representing the ratio between the $G_1$ peak of ovarian cancer cells and the $G_0/G_1$ peak of leukocytes (Barlogie *et al.*, 1983). The percentage of cells in the cell cycle phases was evaluated in only 73 out of 155 samples (because of the presence of more than one cell population causing overlapping of the cell cycle phases or because the number of cancer cells was too small for a good estimate of the cell cycle phases) and was calculated by the method of Baisch (Baisch *et al.*, 1975). The Mann–Whitney test was used for statistical analysis of the percentage of the cells in the synthesis (S) phase of the cell cycle (Giannangeli *et al.*, 1983). Time on study or time to death was calculated from the day of first surgery to the cut-off date or to death, if this occurred. The life-tables method (Kaplan & Meier, 1958) and the log-rank statistic (Tarone & Ware, 1977) were used respectively to estimate and compare survival curves. In order to evaluate to what extent the probability of a better survival depends on ploidy and/or percentage of cells in S phase and other explanatory variables, a multivariate analysis on survival was also done applying the Cox model (Cox, 1972). In this analysis the main effects of ploidy and/or percentage of cells in S phase and of prognostic variables, as well as the effects of the first order interactions of the latter, were investigated. A stepwise procedure was adopted to select the final most parsimonious model containing the statistically significant sub-sets of variables and interactions.

### Results

The DNA index was evaluated on 155 samples from 101 patients, as in 26 patients it was possible to make more than one DNA analysis. In 34 samples, eight primary tumours, 22 ascitic fluids and four omental metastases, the DNA index was 1.00 (DNA diploid tumours) while in the other 121 samples, 26 primary tumours, 79 ascitic fluids and 16 metastases, the DNA index was between 0.85 and 3.00 (DNA aneuploid tumours). Four were DNA tetraploid tumours and only two had a DNA index lower than the DNA diploid tumours. Figure 1 shows representative DNA histograms of different samples with different DNA index.

As shown in Table II, five patients had more than one aneuploid cell population and were classified as DNA multiclonality (Figure 2); in patient no. 24 there were two cell clones in the primary tumour and in ascitic fluid, with different DNA indices.

As described, from 26 patients DNA content was measured on several samples to check the stability of the DNA index over time and from different lesions. Except for patient no. 5, the DNA index was very stable in different samples (Table III). Patient no. 46 was monitored over 30 months, no. 91 over 5 months.

When the DNA index was correlated with the stage of the primary carcinoma of the ovary according to FIGO, we

**Figure 1** Typical flow cytometric analysis of the DNA content of human ovarian cancer cells. **a**, DNA index = 1.00; **b**, DNA index = 0.90; **c**, DNA index = 1.30; **d**, DNA index = 2.00.

**Table II** DNA multiclonality: the six specimens with neoplastic cells of two different DNA indices

| *Patient no.* | *Samples* | *DNA index* |
|---|---|---|
| 20 | Ascitic fluid | 1.24 and 1.53 |
| 21 | Primary tumour | 1.65 and 1.70 |
| 24 | Primary tumour | 0.85 and 1.60 |
| 24 | Ascitic fluid | 0.85 and 1.60 |
| 39 | Ascitic fluid | 1.42 and 1.68 |
| 101 | Primary tumour | 1.39 and 2.30 |

**Figure 2** Example of DNA multiclonality: the arrows indicate the $G_1$ peaks of different DNA aneuploid tumours with different DNA indices. Between the two is the $G_0/G_1$ peak of normal cells present in the tumour. DNA index = 0.85–1.60.

**Table III** DNA index of ovarian tumour cells obtained from different sites and/or in repeated samplings in the same patient

| *Patient no.* | *Primary tumour* | *DNA index in metastases* | *Ascitic fluid* |
|---|---|---|---|
| 3 | 1.89 | | 1.91 |
| 4 | 1.00 | 1.00 | |
| 5 | 1.15 | | 1.72 (2) |
| 13 | 1.68 | | 1.68 |
| 16 | | | 1.67 (2) |
| 17 | 1.35 | | 1.24 |
| 24 | 0.85–1.60 | | 0.85–1.60 |
| 25 | | | 1.80 (8) |
| 32 | | | 1.30 (2) |
| 33 | | | 1.60 (2) |
| 35 | 2.00 | | 2.00 (2) |
| 39 | | | 1.42–1.68<br>1.68 (9) |
| 40 | 1.60 | 1.60 | |
| 45 | | 1.20 | 1.20 |
| 46 | | 1.00 | 1.00 (4) |
| 49 | 1.20 | 1.20 | 1.20 |
| 51 | 0.90 | | 0.90 |
| 66 | | | 1.00 (2) |
| 67 | 1.50 | | 1.50 |
| 74 | 1.65 | 1.65 | |
| 76 | | | 1.90 (4) |
| 80 | | | 1.60 (2) |
| 81 | 1.75 | | 1.75 |
| 88 | | | 1.00 (2) |
| 91 | | 1.80 | 1.80 (7) |
| 100 | 1.87 | 1.87 | 1.87 |

In parentheses are the number of samples analysed.

found that in stages III and IV there were significantly more DNA aneuploid tumours ($P<0.01$) than DNA diploid tumours: respectively 63 and 19. Not enough data were available on tumours classified as Ia, Ib, Ic and II for statistical analysis; nevertheless 7 out of 8 were DNA diploid.

The DNA index was also correlated with the histological grading of the tumours from 92 patients. As shown in Table IV, poorly differentiated tumours and/or tumours classified as 4/4 according to Broders' criteria were more likely to be DNA aneuploid tumours.

DNA diploid or aneuploid tumours were correlated with the residual tumour size (Table V). Patients with residual tumours smaller than 2 cm had the same number of tumours with DNA diploid or aneuploid content, but in patients where the residual tumours were larger than 2 cm, there were significantly more DNA aneuploid tumours than DNA diploid tumours, 58/16 respectively ($P<0.01$). No clusters were found in DNA index for different histological types.

**Table IV** Distribution of DNA diploid and DNA aneuploid tumours and the degree of differentiation of the tumours

| *Tumour classification* | *DNA diploid tumours (cases)* | *DNA aneuploid tumours (cases)* |
|---|---|---|
| Well differentiated | 4 | 6 |
| Moderately differentiated | 7 | 15 |
| Poorly differentiated | 14 | 41 |
| Broders' 1/4 | 1 | 1 |
| Broders' 2/4 | 6 | 8 |
| Broders' 3/4 | 7 | 22 |
| Broders' 4/4 | 11 | 36 |

**Table V** Distribution of DNA diploid and DNA aneuploid tumours in relation to residual tumour size at first surgery

| | *Residual tumour size (cases)* | |
|---|---|---|
| | *⩽2 cm* | *>2 cm* |
| DNA diploid tumours | 11 | 8 |
| DNA aneuploid tumours | 16 | 58[a] |

[a]$P<0.01$ statistical significance ($\chi^2$ test) in respect to DNA diploid tumours in the group of >2 cm.

The percentage of cells in the S phase of the cell cycle was calculated on 73 out of 155 samples run through flow cytometry. There was a significantly lower proportion of cells in S phase in DNA diploid tumours than in DNA aneuploid ones (median 5.05% *vs* 13.45% respectively; $P<0.01$). This difference was clear cut when comparing DNA diploid and DNA aneuploid cells present in ascitic fluids, with 2.96 and 14.86% of the cells in the S phase respectively ($P<0.01$).

The percentage of cells in the S phase was also correlated with the histological grading of the tumours (Table VI). Poorly differentiated tumours and/or tumours classified as 4/4 according to Broders' criteria seemed to have a higher percentage of cells in the S phase of the cell cycle than moderately or well differentiated tumours or 3/4, 2/4, 1/4 Broders'. The samples studied did not show different percentages of cells in the S phase in relation to the histological type.

Univariate analysis on survival and DNA index was performed on the whole population of 90 patients with stages III and IV (Figure 3). At two years the proportion of

**Table VI** Percentage of cells in the S phase of the cell cycle in relation to the degree of differentiation of the tumours

| *Tumour classification* | *% cells in S phase ± s.d.* |
|---|---|
| Well differentiated tumours | 6.9 ± 4.2 |
| Moderately differentiated tumours | 10.0 ± 8.2 |
| Poorly differentiated tumours | 15.7 ± 7.3 |
| Broders' 1/4 | 7.2 ± 6.7 |
| Broders' 2/4 | 7.1 ± 3.5 |
| Broders' 3/4 | 12.7 ± 9 |
| Broders' 4/4 | 17.0 ± 7.7 |

**Figure 3** Survival curves for patients with stage III and IV with DNA diploid or DNA aneuploid tumours.

long-term survivors was considerably higher in the DNA diploid tumour group: 62% (95% confidence limits 44–79) compared to 32% (95% confidence limits 21–43). This difference, however, was not statistically significant ($P = 0.11$). When results were adjusted for size of residual tumour after first surgery (<2 cm or >2 cm) or FIGO stage (I and II *vs* III and IV), patients with a DNA diploid tumour again had a better survival but the difference was not significant in either subgroup (data not shown).

Analysis of survival according to the percentage of cells in the S phase was possible only in a subset of 68 patients for whom survival data were available. Patients with a high percentage of cells in S phase (>5%) had a survival that was not statistically different from that of patients with a low percentage (Figure 4). As with the DNA content the difference became less recognisable when data were adjusted for independent prognostic variables such as residual tumour size and FIGO stage (data not shown).

**Figure 4** Survival curves for patients with stage III and IV with a high (>5%) or low percentage (<5%) of tumour cells in the S phase of the cell cycle.

To study the concomitant effects of several known prognostic factors on survival and their relationship with the DNA index, multivariate analysis was performed on 79 of the 90 patients with advanced tumour (stages III and IV) for whom all the following variables were available: age, residual tumour size (<2 cm, >2 cm), histological type and gradings. Only stage and histological type had a significant impact on survival. The risk of dying increased by factors of, respectively, 2.4 and 1.9 with stage IV and histotype other than serous. All other variables, including DNA index and residual tumour size, had no additional prognostic value as regards survival.

### Discussion

From 101 ovarian carcinoma patients, 155 samples were studied by flow cytometry and evaluated for DNA content. Thirty-four (22%) were DNA diploid tumours and 121 (78%) were DNA aneuploid tumours, as already observed for this type of tumour (Friedlander *et al.*, 1984*b*,*c*; Hedley *et al.*, 1985; Volm *et al.*, 1985*b*) and other types (Barlogie *et al.*, 1980; Frankfurt *et al.*, 1986; Cornelisse *et al.*, 1987).

Considering the high percentage of DNA diploid ovarian tumours one may wonder whether they are overestimated because of the predominance of normal cells present in the sample analysed by flow cytometry. In order to exclude this possibility, we made a cytological analysis of each sample. An atypical cell population was often mixed with contaminating normal cells (particularly in the ascitic fluids); and the ratio differed from one sample to another. In many DNA diploid tumours, the ratio of normal to atypical cells was in favour of the contaminating normal cells. In none of the DNA diploid tumours analysed, however, was the percentage of tumour cells less than 10%. As we are confident that in our conditions we can detect aneuploid cells even when present in a lower proportion (e.g. in experiments in which we mixed 99% DNA diploid cells and 1% of DNA aneuploid cells, the DNA aneuploid cell population was detectable), we think it is reasonable to drop the hypothesis that the DNA diploid tumours are an artificial result due to the limit of the method used.

In six samples we found more than one cancer cell population with a different DNA index (Table II and Figure 2). This was in agreement with the findings of other groups for this type of tumour (Frankfurt *et al.*, 1984*a*; Rodenburg *et al.*, 1987). The number of cases with DNA multiclonality was too small for assessing whether the presence of different neoplastic cell populations with different DNA indices has any meaning in terms of biological and clinical behaviour of the tumour.

The stability of the DNA index over time and in different lesions, monitored in 25 patients, proved to be the same in primary tumour, metastases and ascitic fluids from the same patients and also when many paracenteses were performed over time (see Table III), indicating some degree of stability of the malignant genoma (Frankfurt *et al.*, 1984*a*,*c*; Volm *et al.*, 1985*a*; Iversen & Skaarland, 1987). The DNA index seems to be a very constant feature in ovarian carcinoma and could be considered one of the markers for defining metastatic cells.

A good correlation was found between DNA index and FIGO stage: patients with stage III and IV more frequently had DNA aneuploid tumours, while patients with stage I and II were more likely to have DNA diploid tumours (Iversen & Laerum, 1985).

That DNA index may be indicative of the degree of malignancy is also suggested by the finding that it was related to the degree of differentiation of the tumour (Table IV). Ploidy distribution in tumour samples was related to the size of the residual tumour in the peritoneal cavity after surgery (Table V). Our data are in agreement with those reported by Rodenburg *et al.* (1987), who found that patients with DNA diploid tumours more frequently had residual tumours smaller than 1.5 cm.

Since the DNA index appears stable in the same tumour over time one can speculate that smaller tumours that can be almost completely eradicated surgically leaving a residual tumour less than 2 cm are intrinsically different, perhaps less malignant, from those that are larger at diagnosis. In other words, the post-surgical residual tumour (< or >2 cm) does not depend only on how early the diagnosis is made but may be inherently associated with the biological properties of the tumour.

DNA diploid tumours had a significantly lower percentage of cells in the S phase than DNA aneuploid tumours, as reported by other authors (Friedlander *et al.*, 1983, 1984*c*; Frankfurt *et al.*, 1984*b*, 1986; Iversen & Skaarland, 1987). Even though the flow cytometric measurement of cells in the S phase does not give information on the kinetic parameters of the duration of the cell cycle phase, it could give an estimate of the proliferative activity of the tumour, as reported by Costa *et al.* (1981), who found a good correlation between the estimation of proliferative activity using flow cytometry and autoradiography.

On the other hand, differences in the mean S phase between DNA diploid and DNA aneuploid tumours could well be affected by the fact that normal cells contained within a tumour may contribute to the lower S phase found in the DNA diploid tumours, and the higher value of the S phase in the DNA aneuploid tumours could to some extent be explained by an overlap of S and $G_2M$ phases of the diploid normal cells (Friedlander *et al.*, 1983).

When compared to the degree of malignancy (Table VI), high S phase values were well correlated with poorly differentiated tumours and with tumours classified as Broders' 4/4, suggesting that flow cytometric measurement of the S phase may provide confirmation of the cytological gradings in defining the degree of malignancy in these tumours.

In contrast to reports by Friedlander and other groups (Friedlander *et al.*, 1984*b*; Rodenburg *et al.*, 1987) but in agreement with others (Hedley *et al.*, 1985; Frankfurt *et al.*, 1986), we found that the DNA index was not of major importance as a prognostic factor in ovarian carcinoma. Patients with DNA diploid tumours survived longer than patients with DNA aneuploid tumours, but the difference was not statistically significant, nor was it confirmed by Cox analysis, which corrects for all other recognised prognostic factors in ovarian cancer. Patients with a higher percentage of cells in S phase survived longer than patients with a lower percentage of S phase cells (Figure 4), but not significantly, as Cox analysis did show.

In contrast to the large knowledge existing of many other human tumours, for ovarian cancers not much information is available on the relationship between the proportion of S phase cancer cells and survival of the patients. In the present study we did not find any statistical difference in the survival of patients with a low (5%) or high (>5%) fraction of cells in S phase. These data are essentially consistent with those reported previously by Volm *et al.* (1985) who found that 10 patients with a S-G2M fraction smaller than 17% did not survive longer than 14 patients with a larger percentage of cells in S-G2M phase. Also Frankfurt did not find significant influence of S phase index for the survival of patients in 35 cases of DNA aneuploid tumours (Frankfurt *et al.*, 1986).

Direct comparison of our data with the results of similar studies is extremely difficult because of heterogeneity in the choice of the variables used to fit the regression model, and the different numerical distribution of the patients when stratified by those variables. Our analysis studied the combined impact on survival of ploidy, tumour size, FIGO staging, grading, histotype and age. Other authors (Rodenburg *et al.*, 1987) considered the presence of ascites, peritoneal carcinosis and size of metastasis in addition but excluded age. Among these factors stage and residual tumour size after first surgery are probably the most important. In our series we were unable to find any prognostic significance for tumour size, which in a similar analysis conducted in a much larger population of >500 patients (GICOG, 1987) was the most important determinant for prognosis. Possibly our small sample and the larger number of variables tested may have resulted in an unrealistic definition of apparent correlations. In populations studied by other authors, not larger than ours, conventionally recognised determinants, such as tumour size, also lose their prognostic value. Thus the discrepancy in results is likely to be due to the relatively small series of each study and prospectively collected data should be accumulated before assigning the DNA index a major role as a biological prognostic factor in ovarian cancer.

The generous contribution of the Italian Association for Cancer Research, Milan, Italy, is gratefully acknowledged. This work was supported by the CNR (National Research Council, Rome, Italy), contract no. 85.02209.44.

### References

BAISCH, H. GOHDE, W. & LINDEN, W.A. (1975). Analysis of PCP-data to determine the fraction of cells in the various phases of cell cycle. *Radiat. Environ. Biophys.*, **12**, 31.

BARLOGIE, B. DREWINKO, B., SCHUMANN, J. and 5 others (1980). Cellular DNA content as a marker of neoplasia in man. *Am. J. Med.*, **69**, 195.

BARLOGIE, B., RABER, M.N., SCHUMANN, J. and 6 others (1983). Flow cytometry in clinical cancer research. *Cancer Res.*, **43**, 3982.

COON, J.S., LANDAY, A.L. & WEINSTEIN, R.S. (1987). Advances in flow cytometry for diagnostic pathology. *Lab. Invest.*, **57**, 453.

CORNELISSE, C.J., VAN DE VELDE, C.J.H., CASPERS, R.J.C., MOOLENAAR, A.J. & HERMANS, J. (1987). DNA ploidy and survival in breast cancer patients. *Cytometry*, **8**, 225

COSTA, A., MAZZINI, G., DEL BINO, G. & SILVESTRINI, R. (1981). DNA content and kinetic characteristics of non-Hodgkin's lymphoma: determined by flow cytometry and autoradiography. *Cytometry*, **2**, 185.

COULSON, P.B., THORNTHWAITE, J.T., WOLLEY, T.W., SUGARBAKER, E.V. & SECKINGER, D. (1984). Prognostic indicators including DNA histogram type, receptor content, and staging related to human breast cancer patient survival. *Cancer Res.*, **44**, 4187.

COX, D.R. (1972). Regression models and life tables. *J. Stat. Soc. (B)*, **34**, 187.

ERBA, E., VAGHI, M., PEPE, S. and 6 others (1985). DNA index of ovarian carcinomas from 56 patients: *in vivo/in vitro* studies. *Br. J. Cancer*, **52**, 565.

FRANKFURT, O.S., GRECO, W.R., SLOCUM, H.K. and 4 others (1984*b*). Proliferative characteristics of primary and metastatic human solid tumors by DNA flow cytometry. *Cytometry*, **5**, 629.

FRANKFURT, O.S., SLOCUM, H.K., RUSTUM, Y.M. and 6 others (1984*a*). Flow cytometric analysis of DNA aneuploidy in primary and metastatic human solid tumors. *Cytometry*, **5**, 71.

FRANKFURT, O.S., ARBUCK, S.G., CHIN, J.L. and 7 others (1986). Prognostic applications of DNA flow cytometry for human solid tumors. *Ann. NY Acad. Sci.*, **468**, 276.

FRIEDLANDER, M.L., TAYLOR, I.W., RUSSELL, P., MUSGROVE, E.A., HEDLEY, D.H. & TATTERSALL, M.H.N. (1983). Ploidy as a prognostic factor in ovarian cancer. *Int. J. Gynecol. Pathol.*, **2**, 55.

FRIEDLANDER, M.L., HEDLEY, D.W. & TAYLOR, I.W. (1984*a*). Clinical and biological significance of aneuploidy in human tumours. *J. Clin. Pathol.*, **37**, 961.

FRIEDLANDER, M.L., HEDLEY, D.W., TAYLOR, I.W., RUSSEL, P., COATES, A.S. & TATTERSALL, M.H.N. (1984*b*). Influence of cellular DNA content on survival in advanced ovarian cancer. *Cancer Res.*, **44**, 397.

FRIEDLANDER, M.L., TAYLOR, I.W., RUSSELL, P. & TATTERSALL, M.H.N. (1984*c*). Cellular DNA content – a stable feature in epithelial ovarian cancer. *Br. J. Cancer*, **49**, 173.

GIANNANGELI, A., RECCHIA, M. & ROCCHETTI, M. (1983). SPBS: programs for non parametric tests. *Comput. Programs Biomed.*, **16**, 43.

GRUPPO INTEREGIONALE COOPERATIVO ONCOLOGICO GINECOLOGIA (GICOG) (1987). Randomised comparison of cisplatin with cyclophosphamide/cisplatin and with cyclophosphamide/doxorubicin/cisplatin in advanced ovarian cancer. *Lancet*, **ii**, 353.

HEDLEY, D.W., FRIEDLANDER, M.L. & TAYLOR, I.W. (1985). Application of DNA flow cytometry to paraffin-embedded archival material for the study of aneuploidy and its clinical significance. *Cytometry*, **6**, 327.

IVERSEN, O.E. & LAERUM, O.D. (1985). Ploidy disturbances in endometrial and ovarian carcinomas. A review. *Anal. Quant. Cytol. Histol.*, **7**, 327.

IVERSEN, O.E. & SKAARLAND, E. (1987). Ploidy assessment of benign and malignant ovarian tumors by flow cytometry. A clinicopathologic study. *Cancer*, **60**, 82.

JOHNSON, T.S., WILLIAMSON, K.D., CRAMER, M.M. & PETERS, L.J. (1985). Flow cytometric analysis of head and neck carcinoma DNA index and S-fraction from paraffin-embedded sections: comparison with malignancy grading. *Cytometry*, **6**, 461.

KAPLAN, E.L. & MEIER, P. (1958). Non parametric estimation from incomplete observation. *J. Am. Stat. Assoc.*, **53**, 457.

LAERUM, O.D. & FARSUND, T. (1981). Clinical application of flow cytometry: a review. *Cytometry*, **2**, 1.

LONG, M.E. & SOMMERS, S.C. (1969). Staging, grading and histochemistry of ovarian epithelial tumors. *Clin. Obstet. Gynecol.*, **12**, 937.

OZOLS, R.F., GARVIN, J., COSTA, J. SIMON, R.M. & YOUNG, R. (1980). Advanced ovarian cancer. Correlation of histologic grade with response to therapy and survival. *Cancer*, **45**, 572.

RODENBURG, C.J., CORNELISSE, C.J., HEINTZ, P.A.M., HERMANS, J. & FLEUREN, G.J. (1987). Tumor ploidy as a major prognostic factor in advanced ovarian cancer. *Cancer*, **59**, 317.

TARONE, R.E. & WARE, J. (1977). On distribution-free tests for equality of survival distributions. *Biometrika*, **64**, 156.

VOLM, M., BRUGGEMANN, A., GUNTHER, M., KLEINE, W., PFEIDERER, A. & VOGT-SCHADEN, M. (1985*b*). Prognostic relevance of ploidy, proliferation, and resistance-predictive tests in ovarian carcinoma. *Cancer Res.*, **45**, 5180.

VOLM, M., MATTERN, J., SONKA, J., VOGT-SCHADEN, M. & WAYSS, K. (1985*a*). DNA distribution in non-small-cell lung carcinomas and its relationship to clinical behavior. *Cytometry*, **6**, 348.

YOUNG, R., KNAPP, R.C. & PEREZ, C.A. (1982). Cancer of the ovary. In *Cancer: Principles and Practice of Oncology*, De Vita, V.T., Hellman, S. & Rosenberg, S.A.(eds) p. 884. J.B. Lippincott: Philadelphia.

ZIMMERMAN, P.V., HAWSON, G.A.T., BINT, M.H. & PARSONS, P.G. (1987). Ploidy as a prognostic determinant in surgically treated lung cancer. *Lancet*, **ii**, 530.